# Adenoid Cystic Carcinoma of Mandible

**DOI:** 10.1590/S1808-86942011000600021

**Published:** 2015-10-19

**Authors:** Thiago de Santana Santos, Daniela Guimarães de Melo, Ana Cláudia Amorim Gomes, Emanuel Sávio de Souza Andrade, Emanuel Dias de Oliveira e Silva

**Affiliations:** 1 Specialist in oral and maxillofacial surgery and trauma, Brazilian College of Oral and Maxillofacial Surgery and Trauma. Master's degree in oral and maxillofacial surgery and trauma, Pernambuco Dentistry School (FOP/UPE); 2 Specialist in oral and maxillofacial surgery and trauma, Pernambuco Dentistry School (FOP/UPE). Master's degree student in oral and maxillofacial surgery and trauma, Pernambuco Dentistry School (FOP/UPE); 3 Doctoral degree in oral and maxillofacial surgery and trauma, Pernambuco Dentistry School (FOP/UPE). Adjunct professor of oral and maxillofacial surgery and trauma, Pernambuco Dentistry School (FOP/UPE); 4 Doctoral degree in oral pathology, Rio Grande do Norte Federal University (Universidade Federal do Rio Grande do Norte or UFRN). Adjunct professor of oral pathology, Pernambuco Dentistry School (FOP/UPE); 5 Specialist in oral and maxillofacial surgery and trauma, head of the residence program in oral and maxillofacial surgery and trauma, Oswaldo Cruz University Hospital - HUOC/UPE

**Keywords:** adenoid cystic, carcinoma, medical oncology, pathology, oral

## INTRODUCTION

Malignant neoplasms of the salivary glands are relatively uncommon; they account for less than 7% of head and neck cancers. Of these, about 10% have been diagnosed as the cystic adenoid carcinoma (CAC)^1^. This tumor is thought to originate from segment cells of the intercalar duct or the terminal tubular complex^2^. It commonly affects subjects between the fifth and seventh decades of life, and is closely related with smoking and alcohol intake. There is no racial preference, but the tumor affects mostly women. It typically grows slowly that usually presents clinically as a hard nodule or enlarged mass covered by intact mucosa^3^.

## CASE REPORT

**Figure 1:**
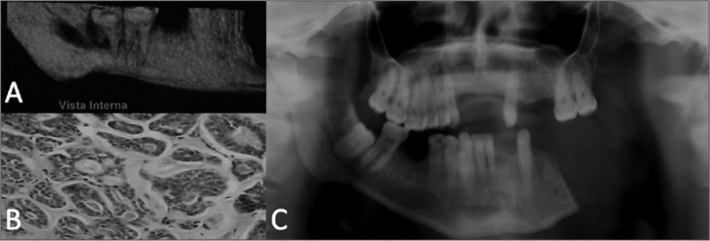
Cystic adenoid carcinoma (CAC): a) 3D reconstructed computed tomography showing osteolysis on the lingual side; c) histopathology showing islands of hyperchromatic cells forming cribriform structures in which hyaline material surrounds tumor cells (HE 200x); c) post-operative panoramic radiogram.

A brown male patient aged 48 years reported moderate pain in the left lower molar region for the past two months. The physical examination of the face revealed no facial asymmetry and enlarged local lymph nodes. On the intraoral examination, the vestibular mucosa along the left lower molars was normal and there was no mass. The oropharynx was hyperemic, and the mucosa was blackened along the lateral portion of the tongue; this area was somewhat hardened and painful upon palpation. Orthopantomography revealed a radiolucent image in the periapical areas of the left lower second and third molars involving the mandibulary canal. Cone beam computed tomography revealed unclear borders, cortical tongue erosion, root resorption of the teeth in question, and involvement of the mandibulary canal (Figure 1A). Surgery showed a solid tumor with unclear borders and low density that compressed the inferior alveolar nerve. The sample was sent to the laboratory and the histopathologic diagnosis was low grade polymorphic carcinoma (Figure 1B). Because of the difficulty in making a differential diagnosis between this lesion and the CAC, and the repercussion on disease progression, immunohistochemical analysis was done; the result was solid CAC. The patient was sent to the oncology department for radical surgery (hemimandibulectomy) and adjuvant radiotherapy (Figure 1C).

## DISCUSSION

The incidence of CAC varies according to the site. It comprises about 1.6% to 2.2% of all tumors and 16% of malignant tumors of the parotid glands. In the submandibular gland this percentage is 12% to 17%; and in the sublingual gland this percentage is 40% of all tumors^3^.

The CAC may present as a slowly growing asymptomatic tumor, although in most cases there are palpation-induced painful events due in theory to the fact that this tumor is markedly neurotropic. In the present case the patient had moderate pain in the lower molar region, especially upon palpation. Perineural invasion is a common finding in this disease, but is not a pathognomonic factor, as it arises in other salivary gland neoplasms such as in low grade polymorphic adenocarcinoma^4^. Histopathology in the present case revealed low grade polymorphic adenocarcinoma. Immunohistochemistry to confirm the diagnosis in fact resulted in a diagnosis of CAC.

Radical surgery combined with postoperative radiotherapy has been the treatment of choice according to some authors; this approach appears to reduce recurrence rates significantly compared with surgery alone^4^. This was the treatment in the present case, as the patient had a solid intra-osseous more aggressive tumor. This approach provides for local control but does not necessarily increase survival. Most authors agree that lymphatic neck dissection should be done only in cases where there neck metastases are clinically demonstrated^5^, which was not the case in this patient.

## FINAL COMMENTS

The CAC is a slow growing neoplasm that generally manifests as an enlarged mass covered by intact mucosa; it is painful upon palpation and metastasizes late in the progression of the disease. Radical surgery is recommended, as this approach reduces the likelihood of metastases.
